# Adherence to Physician Recommendations for Surveillance in Opportunistic Colorectal Cancer Screening: The Necessity of Organized Surveillance

**DOI:** 10.1371/journal.pone.0082676

**Published:** 2013-12-06

**Authors:** Christian Stock, Bernd Holleczek, Michael Hoffmeister, Thomas Stolz, Christa Stegmaier, Hermann Brenner

**Affiliations:** 1 Division of Clinical Epidemiology and Aging Research, German Cancer Research Center (DKFZ), Heidelberg, Germany; 2 Institute of Medical Biometry and Informatics, University of Heidelberg, Heidelberg, Germany; 3 Saarland Cancer Registry, Saarbrücken, Germany; 4 Gastroenterologische Schwerpunktpraxis Völklingen, Kreppstraße 3-5, Völklingen, Germany; Sookmyung Women's University, Korea, Republic Of

## Abstract

**Background:**

Limited evidence exists on the utilization of surveillance colonoscopy in colorectal cancer (CRC) screening programs. We assessed adherence to physician recommendations for surveillance in opportunistic CRC screening in Germany.

**Methods:**

A follow-up study of screening colonoscopy participants in 2007-2009 in Saarland, Germany, was conducted using health insurance claims data. Utilization of additional colonoscopies through to 2011 was ascertained. Adherence to surveillance intervals of 3, 6, 12 and 36 months, defined as having had colonoscopy at 2.5 to 4, 5 to 8, 10.5 to 16 and 33 to 48 months, respectively (i.e., tolerating a delay of 33% of each interval) was assessed. Potential predictors of non-adherence were investigated using logistic regression analysis.

**Results:**

A total of 20,058 screening colonoscopy participants were included in the study. Of those with recommended surveillance intervals of 3, 6, 12 and 36 months, 46.5% (95%-confidence interval [CI]: 37.3-55.7%), 38.5% (95%-CI: 29.6-47.3%), 25.4% (95%-CI: 21.2-29.6%) and 28.0% (95%-CI: 25.5-30.5%), respectively, had a subsequent colonoscopy within the specified margins. Old age, longer recommended surveillance interval, not having had polypectomy at screening and negative colonoscopy were statistically significant predictors of non-adherence.

**Conclusion:**

This study suggests frequent non-adherence to physician recommendations for surveillance colonoscopy in community practice. Increased efforts to improve adherence, including introduction of more elements of an organized screening program, seem necessary to assure a high-quality CRC screening process.

## Introduction

For slightly more than a decade, primary screening for colorectal cancer (CRC) by colonoscopy has been recommended and offered to the general population at average risk for CRC in the United States (US) and Germany. In both countries, screening colonoscopy is generally offered on an “opportunistic” basis, i.e. it depends on individuals to request screening or on their health advisors to recommend screening, and individuals are not invited from a centralized register [1].

Post-polypectomy surveillance using colonoscopy is an integral part of the CRC screening process and required after approximately 20-30% of screening colonoscopies. It has been shown to be an effective tool for prevention of CRC if a good compliance is assured [2,3]. Current guidelines in the US and Germany distinguish between low-risk- and high-risk-adenoma situations in which the recommended surveillance interval is 3 years and 5 (to 10) years, respectively [4,5]. A shorter follow-up is needed in case of incomplete removal of adenomas or, independent of screening, due to signs and symptoms of gastrointestinal diseases.

So far, only limited evidence exists on the actual utilization of surveillance colonoscopy in community practice. Studies conducted in the US reported overuse of surveillance colonoscopy particularly among low-risk individuals but also underuse among individuals with advanced findings at screening [6-9]. Some evidence also suggests that physicians tend to recommend colonoscopic surveillance more frequently than necessary according to guidelines [10-13]. Furthermore, the communication between endoscopists, primary care physicians and patients about results of screening colonoscopies and consequences for surveillance may often be suboptimal [11,14]. Very little evidence on the utilization of surveillance colonoscopy in community practice is available from the German healthcare system despite the introduction of colonoscopy as primary screening offer in 2002. Two recent studies suggested frequent utilization of additional colonoscopies along with substantial adenoma yield in the first 3 years after screening colonoscopy [15], and also substantial inter-physician variation in follow-up habits after screening colonoscopy [16].

In this study, we aimed to assess patient adherence to physician recommendations for surveillance in a follow-up period of up to 4 years after screening colonoscopy in the German opportunistic CRC screening program.

## Methods

A follow-up study of screening colonoscopy participants in Saarland, Germany, was conducted using statutory health insurance (SHI) claims and routine screening colonoscopy documentation data.

### Ethics statement

The study was approved by the data protection commissioner of Saarland and by the ethics committees of the University of Heidelberg and the Medical Association of Saarland. Anonymized data routinely collected by health insurances were analyzed. Written informed consent of patients was infeasible and not required by the approving institutional review board.

### Setting

Saarland is a federal state situated in the south-west of Germany with 1.01 million inhabitants in 2012, corresponding to 1.2% of the German population. In 2008, the age- and sex-standardized utilization of screening colonoscopy in Saarland was 2.9% (age range: 55-74 years), which was slightly higher than the average across all federal states in Germany of 2.4% [17].

Screening colonoscopy has been offered to the general population aged ≥55 years since October 2002 in the German SHI system which covers approximately 90% of the general population (similar offers are also made in the private sector) [18]. Only specialist physicians for internal medicine with subspecialization in gastroenterology or specialist physicians for colorectal surgery are entitled to perform screening colonoscopies and to receive reimbursement. They are required to perform at least 200 colonoscopies per year.

### Data sources

This study is based on health insurance claims and screening colonoscopy documentation data that were both routinely collected by the Regional Association of Statutory Health Insurance Physicians in Saarland between 2007 and 2011. The data cover all SHI funds in Saarland and thus reflect provision of services within the SHI system in the entire state.

The screening colonoscopy documentation data used for the present study are required for physician reimbursement and were available in electronic format. Their contents follow a nationwide standard including information on completeness of the examination, macroscopic and histologic findings, polypectomies, acute complications, diagnoses and recommendations for further procedures. This documentation is routinely done for screening colonoscopies only, i.e., it is not available for colonoscopies performed for other indications.

### Eligibility criteria

Individuals insured by the SHI system and living in Saarland who underwent screening colonoscopy between 2007 and 2009 with and without subsequent recommendation for surveillance colonoscopy were eligible for inclusion in the study. Exclusion criteria were: (i) more than 1 screening colonoscopy coded in the claims data (indicates a coding error), (ii) no related screening colonoscopy documentation available, and (iii) CRC detected by the screening colonoscopy (entails special surveillance scheme after initial cancer treatment).

Individuals without recommendation for surveillance were included to obtain information on the “baseline” utilization of further colonoscopy (most likely to be done for signs and symptoms of gastrointestinal disease) for comparison. If no surveillance is required, it is recommended to undergo a second screening colonoscopy after 10 years in the absence of other risk factors [4].

All individuals with screening colonoscopy in 2007 were followed-up for a minimum of 4 years through to 2011. Among individuals with screening colonoscopy in 2008 or 2009, utilization of further colonoscopies was only ascertained if physicians recommended short-term follow-up within ≤12 months in order to allow a minimum follow-up of one year in addition to the surveillance due date.

### Variables

Utilization of colonoscopy was defined by health insurance claims for outpatient services according to the physician’s fee scale (Einheitlicher Bewertungsmaßstab [EBM]): “01741(M)” for screening colonoscopy and “13421(M)”, “13422(M)” for non-screening colonoscopy. Deterministic encryption procedures were applied to personal identifiers (insurance codes) in the claims data and, in an analogous manner, to personal identifiers (insurance codes) in the screening documentation data. The analyst did not have access to personal information in plaintext stored in the insurance database and was unaware of the encryption procedure. Record linkage between multiple insurance claims for colonoscopy as well as between insurance claims for the initial screening colonoscopy and the respective screening colonoscopy documentation was based on anonymized personal identifiers.

Basic demographic characteristics (age, sex), information on indicators of process quality of the screening colonoscopy (sedation, cecal intubation, polypectomy, complications requiring intervention of the endoscopist), findings (number, size and histology of polyps) and recommendations on time to surveillance colonoscopy were drawn from the screening colonoscopy documentation. It is compulsory to provide information on whether and, if applicable, which further diagnostic or therapeutic procedures were recommended. The data did therefore not contain any missing values with respect to recommendations.

Findings at screening were categorized as ‘negative’ (no adenomas, but including hyperplastic polyps), ‘low-risk adenoma’ (1-3 tubular adenomas, each <1cm, only low-grade intraepithelial neoplasia), and ‘high-risk adenoma’ (≥4 tubular adenomas, ≥1 adenoma ≥1 cm, adenoma with tubulo-villous or villous structure, highgrade intraepithelial neoplasia). Note that usually already ≥3 tubular adenomas are considered a high-risk adenoma situation [4,5]. However, ≥3 tubular adenomas cannot be differentiated in the routine screening colonoscopy documentation in Germany.

For individuals for whom surveillance colonoscopy after 3, 6, 12 or 36 months was recommended by physicians, adherence to the recommendation was defined as having had surveillance colonoscopy at 2.5 to 4, 5 to 8, 10.5 to 16 and 33 to 48 months, respectively. Using these margins, a delay of approximately 33% of the length of the recommended surveillance interval was tolerated. The lower margins allow for short periods before the due dates. They are slightly longer with increasing length of the surveillance interval and are to reflect difficulties and inaccuracies in scheduling colonoscopy.

Given that there is no commonly accepted tolerance margin to define adherence to surveillance colonoscopy, the time-periods above were considered as relatively liberal but still reasonable by the authors. Sensitivity analyses assuming different tolerated delays of 20% and 50% of the length recommended surveillance interval were performed additionally.

In an analysis of predictors of non-adherence to surveillance recommendations, non-adherence was defined as not having had colonoscopy within the specified tolerance margins. Individuals with additional colonoscopy prior to the beginning of the tolerance interval were not considered in this analysis, because these earlier than recommended procedures are likely to have been performed for signs and symptoms of gastrointestinal diseases, i.e. other indications than surveillance. Thus, only individuals with colonoscopy in the specified adherence margins and individuals with colonoscopy later or never after were taken into account. As potential predictors of non-adherence to surveillance recommendations, age group (55-64 years, 65-74 years, ≥75 years), sex (female, male), sedation (yes, no), cecal intubation (yes, no), polypectomy (yes, no), complications (yes, no), adenoma findings at screening colonoscopy (negative, low-risk or high-risk adenoma) and length of recommended surveillance intervall (3, 6, 12, 36 months) were assessed.

### Statistical methods

Cumulative percentages of individuals with additional colonoscopy utilization up to 12, 24, 36, and 48 months after screening colonoscopy, and percentages of individuals adherent to recommendations for surveillance after 3, 6, 12, and 36 months, both along with 95%-confidence intervals (CI), were calculated. Among those with recommended surveillance after 36 months, a subgroup analysis according to screening finding and age group was conducted. Multivariate logistic regression was used to assess associations of a set of potential predictors with non-adherence to physician recommendations. First, univariate associations were calculated and statistically significant variables at the 10% level were included in a full, mutually adjusted model. Variable selection was then performed by backwards elimination until all predictor variables were statistically significant at the 5% level. All statistical analyses were performed in SAS 9.2 (SAS Institute Inc., Cary, NC).

The study was approved by the data protection commissioner of Saarland and by the ethics committees of the University of Heidelberg and the Medical Association of Saarland.

## Results

**Figure 1:**
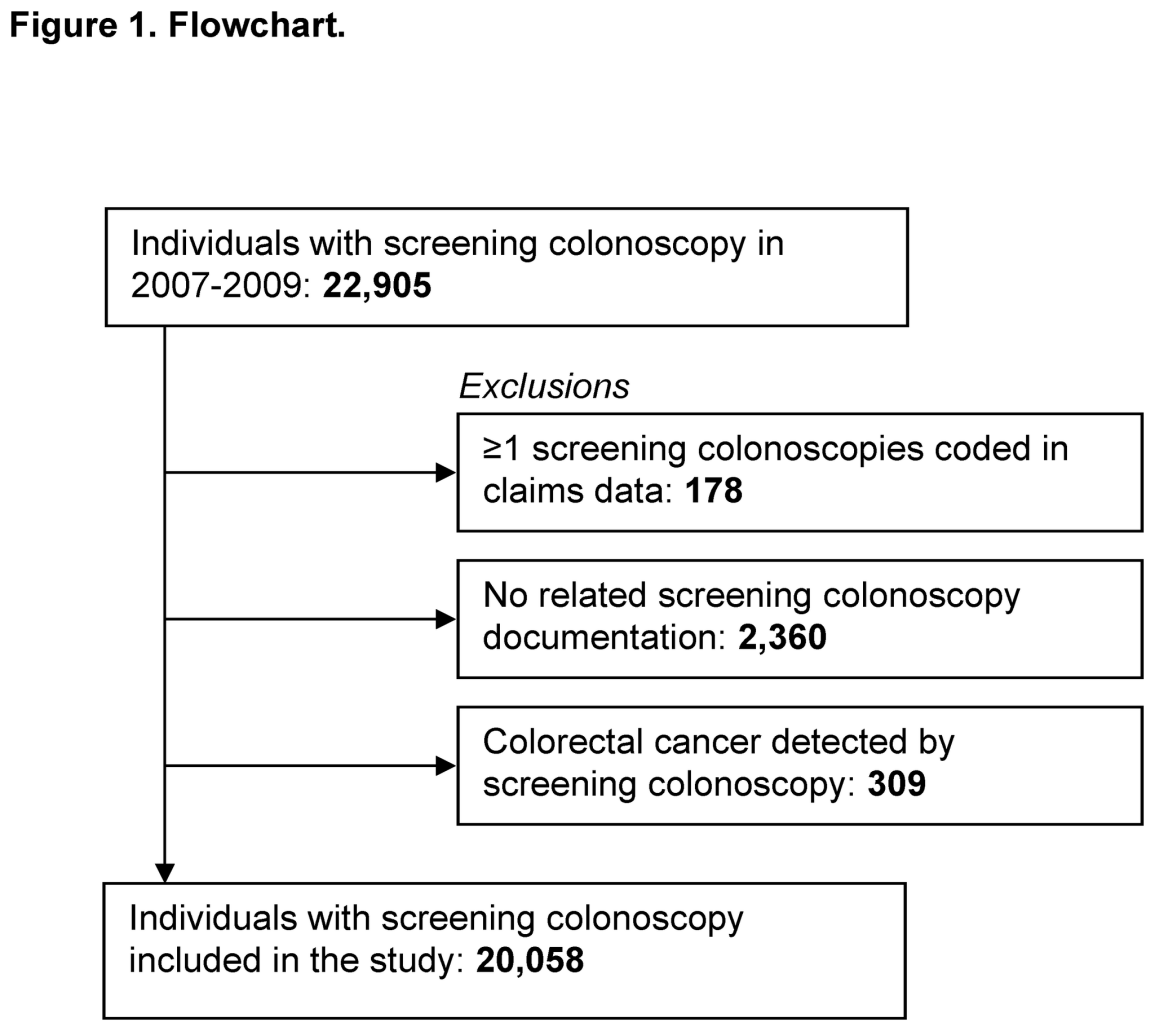
Flowchart.

A total of 20,058 individuals with screening colonoscopies performed between 2007 and 2009 were included in the study. Figure 1 shows a flow diagram of the identification of the study population. Characteristics of the study population are given in Table 1. Slightly more women (54%) than men were included and the mean age was 65 years. An early surveillance colonoscopy after 3, 6 or 12 months was recommended for 3% and surveillance colonoscopy after 36 months was recommended for 18% of the study population. In the majority of screening colonoscopies, 70%, no surveillance (i.e., another screening colonoscopy after 10 years) was recommended.

**Table 1:** Study population.

**Characteristic**	**Year**			**Total**
	**2007**	**2008**	**2009**	
N	6,427	7,140	6,491	20,058
Females	54.3%	54.4%	53.5%	54.1%
Mean age (SD), years	65.0 (7.4)	64.7 (7.5)	64.1 (7.5)	64.6 (7.4)
Age group				
- 55-64 years	48.8%	51.7%	55.6%	52.0%
- 65-74 years	39.4%	37.4%	34.5%	37.1%
- ≥75 years	11.7%	11.0%	9.9%	10.9%
Sedation	80.7%	81.7%	81.8%	81.4%
Cecal intubation	98.6%	98.8%	99.2%	98.9%
Polypectomy	40.2%	42.0%	42.4%	41.5%
Diagnosis				
- Negative colonoscopy	70.5%	69.1%	69.6%	69.7%
- Low-risk adenoma	18.7%	20.6%	20.4%	19.9%
- High-risk adenoma	10.8%	10.3%	10.0%	10.4%
Physician recommendation for surveillance colonoscopy				
- 3 months	0.6%	0.4%	0.7%	0.6%
- 6 months	0.3%	0.5%	0.9%	0.6%
- 12 months	1.8%	2.2%	2.1%	2.1%
- 36 months	19.1%	18.9%	15.1%	17.7%
- Other interval	4.4%	9.7%	14.6%	9.6%
- No surveillance**^*a*^**	73.8%	68.2%	66.6%	69.5%

^a^ Implies recommendation for further screening colonoscopy after 10 years.

Abbreviation: SD, standard deviation.

**Figure 2:**
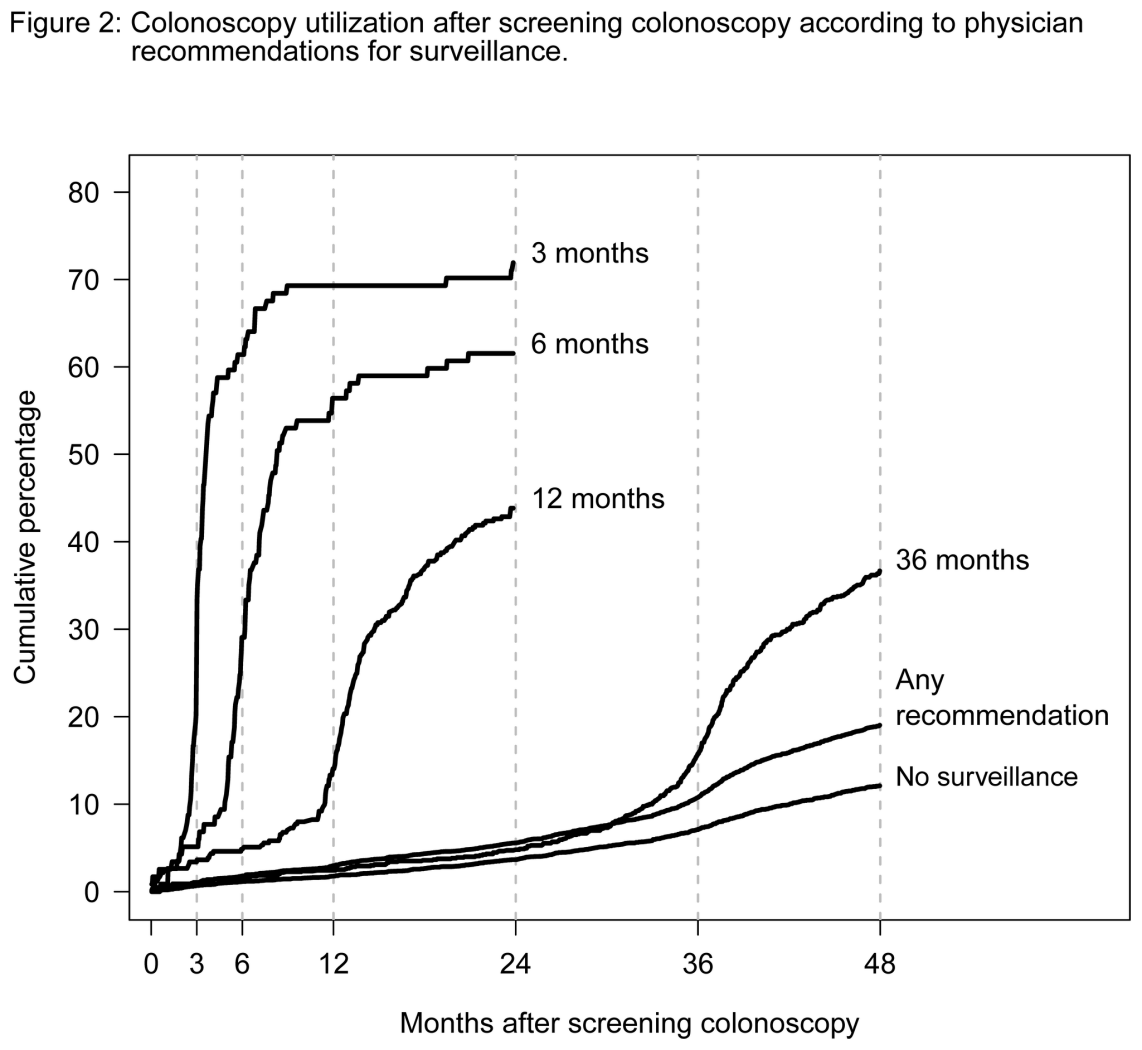
Colonoscopy utilization after screening colonoscopy according to physician recommendations for surveillance.

The cumulative utilization of follow-up colonoscopy according to different surveillance recommendations up to 4 years after the screening colonoscopy is depicted in Figure 2. Utilization started to increase markedly shortly before surveillance was due. The increase was sharper with shorter intervals. Among individuals for whom surveillance after 3, 6 and 12 months was recommended, 72%, 62% and 44%, respectively, had a follow-up colonoscopy within the subsequent 2 years after screening, as shown in Table 2. Of those with recommended surveillance after 36 months, 36% had a follow-up colonoscopy until 1 year after the due date. In this subgroup, the cumulative utilization of follow-up colonoscopy up to 4 years varied slightly depending on whether low-risk adenomas (35%) or high-risk adenomas (43%) had been detected (sensitivity analysis not shown in Tables or Figures). However, substantial variation was observed in the age-groups 55-64 years (42%), 65-74 years (35%) and ≥75 years (17%) (sensitivity analysis not shown in Tables or Figures). Of those without surveillance recommendation, 7% had an additional colonoscopy within 3 years and 12% had an additional colonoscopy within 4 years after screening colonoscopy.

**Table 2:** Utilization of additional colonoscopies up to 4 years after screening colonoscopy.

**Physician recommendationfor surveillance colonoscopy**	**N**	**Cumulative utilization by time after screening colonoscopy**				
		**6 months**	**12 months**	**24 months**	**36 months**	**48 months**
		**% (95% CI)**	**% (95% CI)**	**% (95% CI)**	**% (95% CI)**	**% (95% CI)**
3 months	114	61.4 (52.3, 70.5)	69.3 (60.7, 77.9)	71.9 (63.6, 80.3)	−	−
6 months	117	29.1 (20.7, 37.4)	56.4 (47.3, 65.5)	61.5 (52.6, 70.5)	−	−
12 months	413	4.8 (2.8, 6.9)	14.0 (10.7, 17.4)	43.8 (39.0, 48.6)	−	−
36 months	1,230**^*a*^**	1.6 (0.9, 2.3)	2.4 (1.6, 3.3)	4.7 (3.5, 5.9)	15.4 (13.3, 17.4)	36.3 (33.6, 39.0)
No surveillance**^*b*^**	4,742**^*a*^**	1.2 (0.9, 1.5)	1.8 (1.4, 2.1)	3.6 (3.1, 4.2)	7.0 (6.3, 7.8)	12.1 (11.1, 13.0)
Any recommendation	6,427**^*a*^**	1.8 (1.5, 2.1)	3.0 (2.5, 3.4)	5.6 (5.0, 6.1)	10.7 (9.9, 11.4)	18.9 (18.0, 19.9)

^a^ Only individuals with screening colonoscopy in 2007.

^b^ Implies recommendations for further screening colonoscopy after 10 years.

Abbreviation: CI, confidence interval.Levels of adherence to recommended surveillance intervals of 3, 6, 12 and 36 months according to 3 different definitions of adherence are shown in Table 3. When margins were imposed which tolerate a delay of 33% of the length of the recommended surveillance interval, only less than half of the individuals were considered adherent to surveillance recommendations. Adherence was generally lower with increasing length of the surveillance interval. For different margins with tolerance of delays of 20% and 50% (sensitivity analyses) of the recommended intervals, adherence estimates varied by approximately 10 percentage points.

**Table 3:** Adherence to physician recommendations for surveillance colonoscopy.

**Physician recommendationfor surveillance colonoscopy**	**N**	**Adherence assuming different tolerated delays**		
		**+20% ofsurveillance intervall**	**+33% ofsurveillance intervall**	**+50% ofsurveillance intervall**
		**% (95% CI)**	**% (95% CI)**	**% (95% CI)**
3 months	114	[-2 weeks, +2 weeks]	[-2 weeks, +4 weeks]	[-2 weeks, +6 weeks]
		37.7 (28.8, 46.6)	46.5 (37.3, 55.7)	50.9 (41.7, 60.1)
6 months	117	[-4 weeks, +5 weeks]	[-4 weeks, +9 weeks]	[-4 weeks, +13 weeks]
		30.7 (22.4, 39.1)	38.5 (29.6, 47.3)	42.7 (33.8, 51.7)
12 months	413	[-6 weeks, +10 weeks]	[-6 weeks, +17 weeks]	[-6 weeks, +26 weeks]
		22.3 (18.3, 26.3)	25.4 (21.2, 29.6)	31.2 (26.7, 35.7)
36 months	1,230**^*a*^**	[-12 weeks, +31 weeks]	[-12 weeks, +52 weeks]	[-12 weeks, +78 weeks]
		22.6 (20.3, 25.0)	28.0 (25.5, 30.5)	−

The lower and upper limits of the tolerance intervals are relative to the recommended surveillance dates.

^a^ Only individuals with screening colonoscopy in 2007.

Abbreviation: CI, confidence interval.

The associations between non-adherence to physician recommendations for surveillance colonoscopy (assuming margins that tolerate a delay of 33%) and a set of potential predictors are shown in Table 4. Older age and length of surveillance interval were most strongly associated with non-adherence. In the age group ≥75 years, the odds of non-adherence was increased more than 3-fold compared to age group 55-65 years. Non-adherence was increased more than 2-fold in case of a surveillance interval of 36 months, as opposed to a short-term interval of 3 months. Furthermore, non-adherence was more frequent if no polypectomy had been performed at screening and if the screening colonoscopy was negative for adenomas.

**Table 4:** Predictors of non-adherence to physician recommendations for surveillance colonoscopy.

**Characteristic**		**N**	**Non-adherent^a^**		**Univariate associations**			**Multivariate logistic regression models**				
								**Full model^b^**			**Variable selection^c^**	
					**OR (95% CI)**	***P***		**OR (95% CI)**	***P***		**OR (95% CI)**	***P***
Age group												
	55-64 years	804	64.6%		1.00 Ref.	<0.0001		1.00 Ref.	<0.0001		1.00 Ref.	<0.0001
	65-74 years	695	70.2%		1.30 (1.04, 1.61)			1.29 (1.03, 1.61)			1.30 (1.04, 1.62)	
	≥75 years	196	90.0%		3.29 (2.15, 5.04)			3.43 (2.22, 5.29)			3.46 (2.25, 5.33)	
Sex												
	Female	699	69.7%		1.00 Ref.	0.79						
	Male	996	69.1%		0.97 (0.79, 1.20)							
Sedation												
	Yes	1,328	70.3%		1.00 Ref.	0.09		1.00 Ref.	0.28			
	No	367	65.7%		0.81 (0.63, 1.03)			0.87 (0.68, 1.12)				
Cecal intubation												
	Yes	1,683	69.2%		1.00 Ref.	0.13						
	No	12	91.7%		4.9 (0.63, 38.08)							
Polypectomy												
	No	78	87.2%		1.00 Ref.	0.0009		1.00 Ref.	0.04		1.00 Ref.	0.04
	Yes	1,617	68.5%		0.32 (0.16, 0.63)			0.48 (0.23, 0.98)			0.46 (0.22, 0.95)	
Complications												
	No	1,675	69.2%		1.00 Ref.	0.30						
	Yes	20	80.0%		1.78 (0.59, 5.34)							
Adenoma findings												
	Negative colonoscopy	174	80.5%		1.00 Ref.	0.0001		1.00 Ref.	0.009		1.00 Ref.	0.009
	Low-risk adenoma	872	70.8%		0.59 (0.39, 0.88)			0.62 (0.40, 0.96)			0.62 (0.41, 0.96)	
	High-risk adenoma	649	64.4%		0.44 (0.29, 0.66)			0.52 (0.34, 0.80)			0.52 (0.34, 0.80)	
Recommended surveillance intervall												
	3 months	104	50.0%		1.00 Ref.	<0.0001		1.00 Ref.	<0.0001		1.00 Ref.	<0.0001
	6 months	104	57.7%		1.36 (0.79, 2.36)			1.45 (0.83, 2.55)			1.43 (0.82, 2.51)	
	12 months	380	73.7%		2.80 (1.79, 4.38)			2.64 (1.66, 4.20)			2.61 (1.64, 4.15)	
	36 months	1,107	70.7%		2.42 (1.61, 3.63)			2.48 (1.61, 3.83)			2.47 (1.60, 3.82)	

^a^ Non-adherence was defined as not having had additional colonoscopy in the time-period from 2, 4, 6, or 12 weeks prior to the recommended surveillance date to 4, 9, 17, or 52 weeks after the recommended surveillance date in case of surveillance recommendations of 3, 6, 12 and 36 months, respectively. Using this definition, individuals may exceed the recommended surveillance interval by up to +33% to still be considered adherent to surveillance recommendations. Of the overall 1,874 individuals with surveillance recommendations of 3, 6, 12, or 36 months, 179 individuals with early repeat colonoscopy before the tolerance interval were excluded from this analysis as such colonoscopies are unlikely to have been performed for surveillance.

^b^ All variables who were statistically significantly associated with non-adherence at a significance level 10% in univariate models were considered in the full model.

^c^ Variable selection by backwards elimination was performed until all variables in the model were statistically significantly associated with non-adherence at significance level 5%.

Abbreviations: CI, confidence interval; OR, odds ratio; Ref, reference.

## Discussion

In this study, screening colonoscopy participants in the German “opportunistic” CRC screening program were followed-up for utilization of additional colonoscopies for up to 4 years using statutory health insurance claims data. Colonoscopy utilization started to increase shortly before recommended surveillance colonoscopies were due, but the cumulative percentages of colonoscopy utilization were still low a year after the recommended surveillance dates. For the different recommended intervals and definitions of adherence commonly only less than 50% of participants could be considered adherent to surveillance recommendations. Thus, surveillance was often either not utilized or only with considerable delay.

Non-adherence was more pronounced with increasing length of the surveillance interval. An extension of the surveillance interval in the low-risk adenoma situation from 3 to 5 years in 2008 could partially explain the low levels of adherence among participants with screen-detected low-risk adenomas [19]. However, as a subgroup analysis showed, adherence was also low among participants with high-risk adenomas. Furthermore, non-adherence was more pronounced with high age (especially ≥75 years). This observation may partly be explained by increasing age-related comorbidities (along with increased risk of adverse effects) and immobility in the elderly population which could hamper colonoscopy utilization. Concerning the stopping of surveillance, the 'European guidelines for quality assurance in colorectal cancer screening and diagnosis' state that the decision for or against a surveillance examination "should depend not only on adenoma characteristics, but also on the patient’s age and wishes, and the presence of significant co-morbidity" [20]. Furthermore, negative screening colonoscopy and screening colonoscopy without polypectomy were also positively associated with non-adherence to surveillance recommendations, but these subgroups were small and it seems likely that other reasons than adenoma surveillance could have led to the recommendation of a follow-up, e.g. repeat colonoscopy due to inadequate bowel preparation.

Significant waiting times for colonoscopy might in theory cause patients to be falsely classified as non-adherent to surveillance. However, with respect to the German healthcare system, it would be exceptional if a surveillance colonoscopy could not be performed within a short time (presumably less than a month) after contacting a gastroenterologist’s office. Problems to schedule a colonoscopy are therefore unlikely to have a major impact on the results.

Most of the published evidence on surveillance colonoscopy utilization stems from the US healthcare system. It suggests that rates of follow-up colonoscopies may be higher in the US than in the present German study, although comparability is limited due to different outcome measures [7,8,21,22]. Based on data from the late 1990s, the fraction of colonoscopies done for adenoma surveillance in the US was reported to be 24%, whereas it was only 11% in Bavaria, Germany, in 2006 [23,24]. A recent study from the German healthcare system suggested utilization rates of additional colonoscopies in the same magnitude as observed in the present study, but did not assess adherence to recommendations [15]. It could only track patients if they returned to the same gastroenterologist who performed screeening, whereas here any further colonoscopies irrespective of the provider could be taken into account. Another study, based on the same cohort as the former study, indicated that follow-up habits might vary substantially between physicians [16].

Alerting systems and reminders have been shown to effectively increase adherence to surveillance [25,26]. Given the observed low adherence, it appears sensible to consider their widespread implementation. Furthermore, health services research studies on physician-patient communication concerning screening results and surveillance requirements might help to better explain low levels of adherence to surveillance recommendations and to identify further risk factors for non-adherence, which could be targets for interventions to improve adherence to surveillance.

Apart from surveillance, participation in screening colonoscopy in the German “opportunistic” CRC screening program is also known to be low [18,27]. European guidelines have recently advocated implementation of “organized” CRC screening to ensure high coverage, and initiatives have been started in Germany to prepare personal invitations to CRC screening on a nationwide basis, i.e. to introduce a central element of “organized” screening [28,29]. The present study supports suggestions that colonoscopy screening should be regarded as a “package” that includes surveillance [30]. In forthcoming reforms of the CRC screening system in Germany, which are likely move towards more “organized” forms of screening, it may be of importance to also consider invitations and reminders with respect to surveillance in order to assure a high quality of the CRC screening program overall.

This analysis focused on individual recommendations made by the physicians who performed screening colonoscopy. An assessment of the appropriateness of these recommendations according to guidelines was deemed beyond the scope of this article. Studies conducted in the US healthcare system have suggested that physicians often deviate from guidelines by recommending earlier or unnecessary colonoscopic surveillance, e.g. in case of hyperplastic (i.e. non-adenomatous) polyps [12,31]. In this study, only a very small fraction of participants without adenoma findings had a recommendation for a surveillance colonoscopy suggesting that inappropriate recommendations for surveillance may have been rare in practice.

Several limitations are important to be considered for the interpretation of the present results. First, this study is based on routine data which were not primarily collected for scientific purposes. Several patient-level factors were unavailable that would have been of interest because they possibly impact on adherence to surveillance recommendations, e.g. education, smoking, and obesity. Also, physician-level factors such as experience, colonoscopy volume or specialization were not available but might influence adherence to surveillance colonoscopy. Second, due to a lack of similar studies and no available expert-consensus, the margins chosen to define adherence are subject to a certain degree of arbitrariness. However, even when fairly liberal adherence margins were applied in sensitivity analyses the overall observation of low adherence levels remained unchanged. Third, only surveillance recommendations up to 3 years were considered in the present study. There were very few recommendations for 5 years (classified under ‘other interval’ in Table 1) because at the time when most of the included screening colonoscopies were conducted, the recommended interval was still 3 years both in the high-risk and low-risk adenoma situation [32]. Fourth, possible losses to follow-up due to unobserved mortality might have resulted in an underestimation of surveillance rates. Using age- and sex-specific life-tables of the Saarland population, we estimated that 7% of the study population who had a recommendation for colonoscopic surveillance in 2007 can be expected to have died in the subsequent 4 years. Thus, the potential underestimation of the cumulative utilization in this time-period caused by mortality may be at most 7 percentage points (probably lower because people with severe comorbidity are less likely to have undergone screening colonoscopy). Fifth, it is unknown whether additional colonoscopies were actually performed for surveillance. They may partly also have been done primarily for signs and symptoms of gastrointestinal diseases. The codes used for health insurance claims do not distinguish between diagnostic and surveillance colonoscopies. According to the findings among participants with no surveillance recommendation, up to 12% may have had another colonoscopy within 4 years for diagnostic reasons. Finally, when predictors of non-adherence of surveillance colonoscopy were assessed in logistic regression models, only a small proportion of the evidence was based on individuals with a short-term surveillance recommendation of 3 or 6 months. In additional stratified analyses by recommended interval, the identified predictors of non-adherence (age-group, polypectomy, adenoma findings) did not reach statistical significance for these short-term intervals (results not shown). Therefore, confirmation of the predictors for short-term intervals in larger samples is required.

The results observed in this study reflect community practice in Saarland, Germany, over the time-period from 2007 to 2011. Although this is a regional assessement, due to identical incentive schemes for physicians the results may, in principle, be generalizable to the SHI system in the whole of Germany. Variation is likely to exist between physician practices. Physicians partially invite or remind their patients of the due surveillance colonoscopy, but the extent of this practice is unclear.

In summary, this study suggests frequent non-adherence to physician recommendations for surveillance colonoscopy in community practice, particularly with longer surveillance intervals. Increased efforts to improve adherence, including introduction of more elements of an organized CRC screening program (e.g. widespread use of alerting systems and reminders with invitations to surveillance colonoscopy), seem necessary to assure a high-quality CRC screening process. The upcoming changes to the CRC screening system in Germany in order increase screening uptake and to implement Eurpean guidelines on CRC screening may provide an opportunity to also increase the utilization of surveillance colonoscopy.
